# Quartz-Enhanced Photoacoustic Spectroscopy with Right-Angle Prism

**DOI:** 10.3390/s16020214

**Published:** 2016-02-06

**Authors:** Yongning Liu, Jun Chang, Jie Lian, Zhaojun Liu, Qiang Wang, Zengguang Qin

**Affiliations:** School of Information Science and Engineering, Shandong University, Jinan 250100, China; Liuyongning1990@163.com (Y.L.); lianjie@sdu.edu.cn (J.L.); zhaojunliu@sdu.edu.cn (Z.L.); iamwq1989@gmail.com (Q.W.); qinzengguang@sdu.edu.cn (Z.Q.)

**Keywords:** right-angle prism, QEPAS, gas detection, DFB-LD

## Abstract

A right-angle prism was used to enhance the acoustic signal of a quartz-enhanced photoacoustic spectroscopy (QEPAS) system. The incident laser beam was parallelly inverted by the right-angle prism and passed through the gap between two tuning fork prongs again to produce another acoustic excitation. Correspondingly, two pairs of rigid metal tubes were used as acoustic resonators with resonance enhancement factors of 16 and 12, respectively. The QEPAS signal was enhanced by a factor of 22.4 compared with the original signal, which was acquired without resonators or a prism. In addition, the system noise was reduced a little with double resonators due to the *Q* factor decrease. The signal-to-noise ratio (SNR) was greatly improved. Additionally, a normalized noise equivalent absorption coefficient (NNEA) of 5.8 × 10^−8^ W·cm^−1^·Hz^−1/2^ was achieved for water vapor detection in the atmosphere.

## 1. Introduction

Since its first introduction by Kosterev *et al.* in 2002 [1], quartz-enhanced photoacoustic spectroscopy (QEPAS) has been widely demonstrated for trace gas detection with outstanding performance [2,3]. A commercially available quartz tuning fork is used as a sharp resonant acoustic receiver for its excellent frequency response characteristics. To further enhance the photoacoustic signal, an acoustic microresonator is used to amplify the acoustic signal with a factor of dozens or even more [1,2,3,4,5,6]. Normally, the acoustic resonator (AR) is a pair of two short rigid metal tubes, placed on two sides of tuning fork closely [6], which is called the on-beam structure, or the tubes are placed on a relatively long tube with a small slit in the middle [7], and such a structure is known as an off-beam QEPAS. 

In addition to using ARs to enhance the photoacoustic signal, many other enhancement methods were reported. Borri *et al*. presented an intracavity QEPAS (I-QEPAS) technique for trace gas detection [8,9]; the method combined QEPAS with a buildup optical cavity based on quantum cascade lasers, and it is suitable for cases where a long absorption distance can be achieved by the lens. For some applications, a fiber collimator may be more convenient. However, the valid work distance is very limited when the collimated laser beam needs to successfully pass through the tuning fork prongs. In such a case, Zheng *et al*. utilized two tuning forks which increased the signal intensity with an enhancement factor of 1.6 [10]; they also presented a double-pass QEPAS sensor with a low-cost, high-reflection concave mirror [11]. Dong *et al*. developed a system with double acoustic microresonators and formed double-channel detection with two laser diodes [12]. Inspired by their work, we proposed another configuration for double ARs with a right-angle prism. 

Commonly, the work distance of a collimator ranges from 20 mm to 30 mm. For 32.768 kHz tuning forks, the length of the whole acoustic resonance structure is about 10 mm; usually, after passing through the AR, the laser beam dissipates in the space. Based on that fact, the laser can be better used if the beam can be recycled. In this article, a right-angle prism was used in the light path to invert the laser-propagating direction. After passing through the first AR, the laser beam was inverted, and then the parallel inverted beam excited the target gas again by propagating through the second AR, which is paralleled closely with the first AR. The proposed structure can significantly improve the PA signal compared with the single-pass structure.

In QEPAS, the excitation laser source is modulated at half of the resonance frequency for the selected tuning fork. When the laser beam irradiates on the target gas, the electromagnetic radiation provides vibrational excitation of the molecules by changing the dipole moment. Then the excitation is converted to the translational molecular motion (V-T transfer), which releases heat to the gas [13]. If the relaxation time of the nonradiative transition is less than the modulation period of the incident laser, when gas molecules return back from the excited state to the ground state, the gas temperature will be modulated as well. It implies periodic thermal expansion and pressure wave (acoustic wave) generation. Usually, the original photoacoustic (PA) signal is enhanced by an AR unit to get amplification. The signal amplitude in the AR can be expressed as [14]:

(1) A=C(ω)αW

where *C*(*ω*) denotes the parameter of the AR unit, particularly related to the resonant enhancement factor and modulation angular frequency *ω*; *α* is the absorption coefficient related to the detected gas concentration and absorption line characteristics; *W* is the laser power operating at a specific wavelength overlapping with the target gas absorption line. 

The above enhanced PA signal is detected by the tuning fork, which is placed between the rigid metal tubes of the AR unit. The piezoelectric current is proportional to the PA signal and the *Q* factor of the tuning fork, and its expression can be written as [14]:

(2) $$\begin{array}{ll} S & =kQ\times A\\ & =kC(\omega )Q\alpha W \end{array}$$

where *k* is the piezoelectric conversion coefficient of the tuning fork, *Q* is the quality factor of the tuning fork. 

When a transimpedance amplifier with super-low noise is used, the QEPAS sensor noise is primarily determined by the thermal noise of the used tuning fork. The tuning fork noise measured after the transimpedance amplifier (without further amplification) at the resonance frequency is equal to the thermal noise of the equivalent resistor *R* [2,3]. The noise rms voltage is [5]:

(3) $$\sqrt{\langle V_{N}^{2}\rangle }=R_{g}\sqrt{\frac{4k_{B}T\Delta f}{R}}$$

(4) $$R=\frac{1}{Q}\sqrt{\frac{L}{C}}$$

where *R_g_* is the gain resistor (usually *R_g_* = 10 MΩ). *R_g_* also can introduce noise which is *(R_g_/R)*^1/2^ times lower than the tuning fork noise, and such noise can be neglected because the typical value of *R* is ~10 to 200 kΩ. *T* is the tuning fork temperature, and Δ*f* is the detection bandwidth. For tuning forks, *R* represents the oscillator loss, and is related with the *Q* factor, equivalent inductance *L* and equivalent capacitance *C* of the tuning fork. As *L* and *C* do not change more than 0.1% under interaction with gas [5], the noise of the used tuning fork is proportional to *Q^1/2^*. However, if the noise level for a transimpedance amplifier has reached a few microvolts it should be considered, as the tuning fork noise calculated by Equation (3) is about 3 μV when *R* = 100 kΩ.

## 2. Experiment Setups 

**Figure 1:**
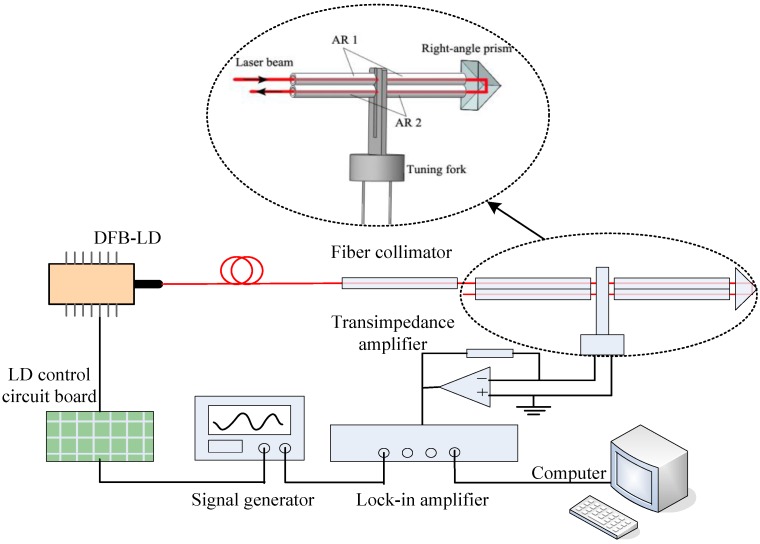
Schematic of the QEPAS system with a right-angle prism.

The schematic diagram of QEPAS system with right-angle prism and two ARs (AR1 and AR2) is shown in Figure 1. The axes of two ARs passed through the gap between two fork prongs. AR1 was located 0.7 mm below the prongs top-ends, and AR2 was paralleled closely with AR1. Tube lengths were measured to be 4.96 mm and 4.98 mm for AR1, respectively, 4.94 mm and 4.96 mm for AR2, with the same inner diameter of 0.48 mm and the same thickness of 0.10 mm. A fiber-coupled distributed feedback laser diode (DFB-LD, Sichuan Tengguang Science and Technology Co., Ltd., Mianyang, China, model WSLS-137010C1424-20) operating at 1368.3 nm was used as the excitation light source generating PA signal, with emission characteristics of 0.11 mW/mA and 0.004 nm/mA. Its tuning spectral range overlapped the water vapor absorption line at 1368.597 nm with a line intensity of 1.8 × 10^−2^ cm^−1^/(molecule·cm^−2^) at 296 K based on the HITRAN database. The DFB-LD injection current and temperature control were provided by a self-developed drive circuit. The laser current was sine wave modulated at half resonant frequency of tuning fork, and the sine wave signal was generated by a signal generator and superimposed with scanning signal by an analog adder on LD control circuit board. The laser beam was collimated by a fiber-coupled collimator (Wuhan six nine Sensing Technology Co., Ltd., Wuhan, China) with 0.25 mm waist spot diameter and 25 mm work distance. A tuning fork (DT-38, 32.768 kHz, 12.5 pF) with exact resonant frequency of f_0_ = 32.755 kHz (3 dB bandwidth is 4 Hz) in atmosphere was selected as acoustic detector. Two double-tube ARs (AR1 and AR2) were used to enhance the PA signals, which were excited by incident laser beam and reflected beam inverted by the right-angle prism (made of K9 glass). The tuning fork-generated piezoelectric current was converted into voltage by a homemade transimpedance amplifier (noise voltage is about 8 μV for *R* = 100 kΩ within 0.51 Hz noise bandwidth) with a gain resistor of 10 MΩ. The signal was demodulated by the lock-in amplifier (Zurich Instruments, model HF2LI) at f_0_. Time constant of the lock-in amplifier was 200 ms in combination with a 12 dB/octave slope filter; namely, the lock-in amplifier bandwidth was 0.51 Hz.

## 3. Experiment Results and Discussion

**Figure 2:**
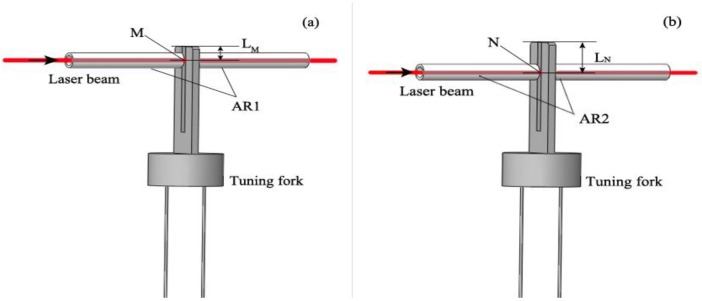
Two ARs work separately; (**a**) the case where only AR1 is used; (**b**) the case where only AR2 is used.

**Figure 3:**
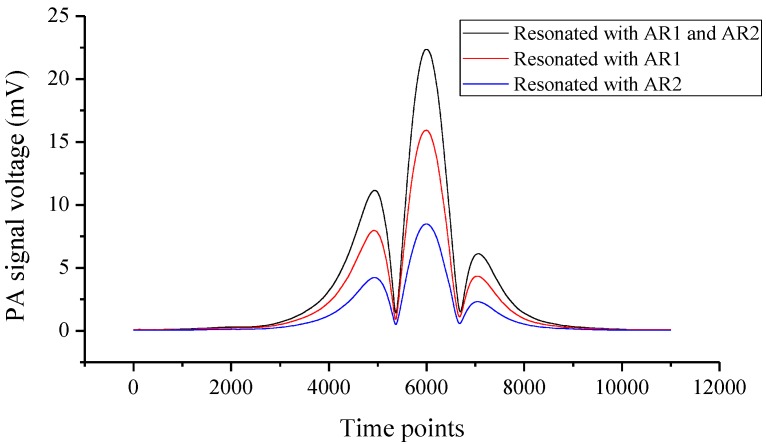
PA signals in different resonant cases; the black line represents the total PA signal resonated by combination of AR1 and AR2; the red line denotes the PA signal only resonated by AR1; the blue line is the PA signal only resonated by AR2.

In the following experiments, the laser scanning cycle was set to 130 s, and the output laser power was 6.8 mW. The experiment was operated in atmosphere at 21.1 °C. The water vapor concentration was 0.83%. The PA signals after the lock-in amplifier were exhibited. We tested the performance of the prism and the two ARs as shown in Figure 1, and we also tested the individual performance of each AR. The resonance structures when the two ARs worked separately are given in Figure 2, and the experiment results are demonstrated in Figure 3. When only resonated by AR1, as Figure 2a shows, the PA signal (15.92 mV) was enhanced by a factor of 16 compared with the original excited PA signal (1.0 mV). M was the optimum incident point for the laser beam, and the distance between the prongs’ top-ends and the laser incident point (M point) was about 0.7 mm (L_M_ ≈ 0.7 mm). When only resonated by AR2, as Figure 2b shows, the PA signal (8.48 mV) was enhanced by a factor of 12 times compared with its original signal (0.72 mV). Here, the distance between the prong’s top-ends and the laser incident point (N point) was about 1.38 mm (L_N_ ≈ 1.38 mm), in which space AR1 and AR2 can be placed closely. The resonance situation only with AR1 in Figure 2a is the most common case in on-beam QEPAS systems. On this basis, a right-angle prism (a size of 2 mm × 2 mm × 2 mm) was used to invert the laser-propagating direction after its first PA excitation. Then the inverted laser beam passed through AR2, and increased the PA signal by a large margin with another PA excitation. Finally, the signal was enhanced to 22.37 mV as Figure 3 shows. As M was the optimum incident point for the laser beam, the system’s original PA signal should be 1.0 mV. The system resonance enhancement factor was improved to 22.4.

**Figure 4:**
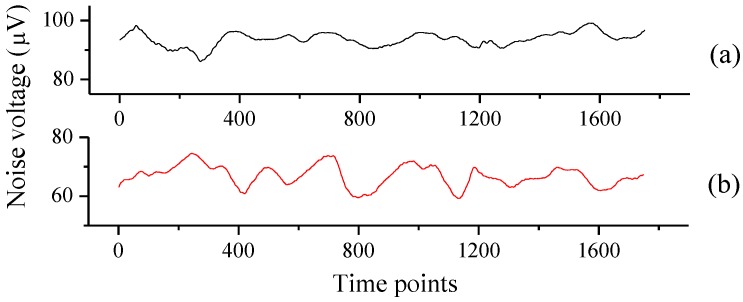
Noise in different resonant conditions; (**a**) system noise when both AR1 and AR2 were used; (**b**) system noise when only AR1 was used.

**Figure 5:**
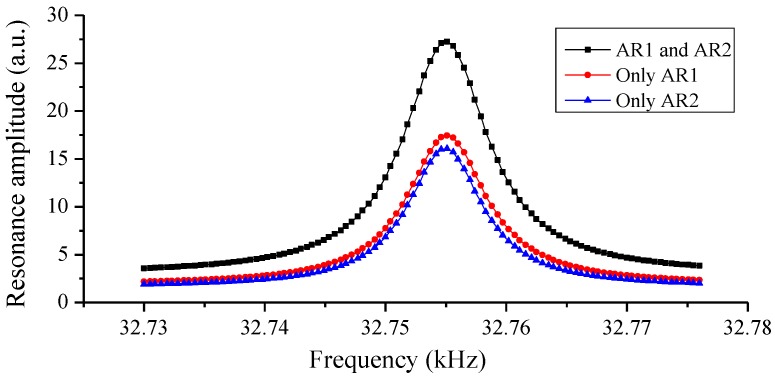
Resonance curves obtained for different configurations; the black square represents the double ARs case; the red dot denotes the case only AR1 was used; the blue triangle is the case AR2 used only.

When only resonated by AR1, the noise was about 14.3 μV. As Figure 4a shows, the system noise was reduced to 11.6 μV after adding the right-angle prism and AR2. Tuning fork noise is proportional to the square root of the *Q* value. The addition of AR2 changed the *Q* factor for the configuration when only AR1 was used. We measured the resonance curves in Figure 5 for different configurations of Figure 1 and Figure 2. It shows that AR1 decreased the *Q* value from 8188 for the bare tuning fork to 6065; the *Q* value was further reduced to 5283 after AR2 was added, due to the stronger coupling effect between the tuning fork and the two ARs.

In the QEPAS system, the detection sensitivity is expressed by the normalized noise equivalent absorption coefficient (NNEA)

(5) $$NNEA=\frac{NSg(\nu )\cdot W}{\sqrt{B}\cdot \frac{V_{s}}{V_{n}}}$$

(6) $$SNR=\frac{V_{s}}{V_{n}}$$

where *N* is the number density of the molecules; *S* is the line strength of the selected absorption peak; *g(ν)* is the gas line shape function; *W* is the laser output power; *B* is the equivalent noise bandwidth, *V_s_* is the PA signal voltage, *V_n_* is the system noise. *SNR* denotes the system signal-to-noise ratio.

A better system SNR is significant for detection sensitivity. The original PA signal generated by gas absorption is weak; moreover, the tuning fork has its detection limit. For trace gas detection, acoustic signal enhancement is particularly important. In the experiment, to make it close for the two ARs, the inner diameter of the used rigid metal tubes should be small. Although the enhancement effect for each AR was not attractive enough, it did not influence the experiment design concept. According to the experiment data, the system SNR has been improved from 1113 to 1928. 

As introduced previously, the output power of the distributed feedback laser diode (DFB-LD) was 6.8 mW. The total insertion loss of the connected flange and the collimator was about 0.3 dB. So the incident laser power for the first acoustic excitation was 6.3 mW. The transmittance of the used prism (K9 glass) at 1370 nm is 92%. Ideally, the incident laser power for the second acoustic excitation should be ~5.8 mW. For Equation (5), *W* = 6.3 mW, *N* = 2.07 × 10^17^ molecule·cm^−3^, *S* = 1.8 × 10^−2^ cm^−1^/(molecule·cm^−2^), *g(ν)* = 3.41 cm at 1368.597 nm in the Lorentz line shape function, *B* = 0.51 Hz. Based on the facts above, the system NNEA should be 5.8 × 10^−8^ W·cm^−1^·Hz^−1/2^, corresponding to a detection sensitivity of 4.3 ppm for water vapor.

## 4. Conclusions

In summary, we proposed and demonstrated a very simple and useful method to enhance the PA signal with two acoustic resonators (ARs). The two ARs took full use of the effective laser beam collimated by a fiber collimator and enhanced the PA signal by superposition. When only resonated by one AR the maximum resonance enhancement factor was 16. A right-angle prism was inserted in the light path to invert the laser propagating direction, and then the inverted laser beam excited the target gas, generating PA signal again. The total enhancement factor could be promoted to 22.4 by adding another AR in the inverted light path. The system total noise was reduced a little due to the *Q* factor decrease. The detection SNR was greatly improved. A NNEA of 5.8 × 10^−8^ W·cm^−1^·Hz^−1/2^ was achieved for water vapor in atmosphere. Moreover, the prism was very small, and it almost did not change the detector volume.
